# Full-Dose Intraoperative Electron Radiotherapy for Early Breast Cancer: Evidence from a Single Center’s Experience

**DOI:** 10.3390/cancers15123239

**Published:** 2023-06-19

**Authors:** Antonio Stefanelli, Eleonora Farina, Edoardo Mastella, Sara Fabbri, Alessandro Turra, Simona Bonazza, Alessandro De Troia, Margherita K. Radica, Paolo Carcoforo

**Affiliations:** 1 Department of Radiation Oncology, University Hospital of Ferrara, 44121 Ferrara, Italy; 2 Department of Medical Physics, University Hospital of Ferrara, 44121 Ferrara, Italy; 3 Department of Surgery, University Hospital of Ferrara, 44121 Ferrara, Italy

**Keywords:** breast cancer, intraoperative radiotherapy, early stage

## Abstract

**Simple Summary:**

A retrospective observational study was proposed to evaluate the clinical response rate and cosmetic outcome after full-dose intraoperative electron radiotherapy (IOERT) in early low-risk breast cancer treated with conserving surgery. 162 patients were included in this analysis (median follow-up: 54 months, range: 1–98 months). IOERT was delivered with a dose of 21 Gy at 90% isodose. The overall response rate was 97.5% (CI 95%: 0.93–0.99%). Locoragional relapse occurred in 2.5% of patients. No patient showed distant metastases. No patient showed radiation-related acute complications, 3.7% of patients late G2–3 toxicity. Only 3.7% of patients showed poor cosmetic results. The results highlighted the safety and effectiveness of the full-dose IOERT treatment for highly selected patients.

**Abstract:**

To evaluate the clinical response rate and cosmetic outcome after full-dose intraoperative electron radiotherapy (IOERT) in early breast cancer (BC) treated with conserving surgery. Inclusion criteria were: >60 years old, clinical tumor size ≤2 cm, luminal A carcinoma, patological negative lymph nodes, excluded lobular carcinoma histology. IOERT was delivered with a dose of 21 Gy at 90% isodose. Clinical, cosmetic and/or instrumental follow-up were performed 45 days after IOERT, 6 months after the first check, and every 12 months thereafter. Acute and late toxicities were assessed with the CTCAE v.4.03 and EORTC-RTOG scales, respectively. Cosmetic outcome was evaluated using the Harvard/NSABO/RTOG Breast Cosmesis Grading Scale. Overall, 162 consecutive patients were included in this analysis (median follow-up: 54 months, range: 1–98 months). The overall response rate was 97.5% (CI 95%: 0.93–0.99%). Locoragional relapse occurred in 2.5% of patients. No patient showed distant metastases. No patient showed radiation-related acute complications, with 3.7% showing late G2–3 toxicity. Only 3.7% of patients showed poor cosmetic results. Our data confirmed that IOERT is a feasible and valid therapeutic option in low-risk BC patients treated with lumpectomy. A low local recurrence rate combined with good cosmetic results validates the settings of our operative method in routinely clinical practice.

## 1. Introduction

Breast cancer (BC) is currently one of the most frequent malignancies worldwide. Approximately 2.3 million new cases are diagnosed globally every year occurring as the fifth cause of overall cancer-related death [1,2]. Currently, breast-conserving surgery is the leading approach, especially for early BC. Radiotherapy (RT) has an established role in the BC multidisciplinary management, reducing local relapse rates in adjuvant setting [3,4,5,6,7,8,9,10,11]. As reported in the literature, intraoperative (IO)RT is a valid therapeutic option for women with BC at low risk of recurrence [4,5,6,7,8,9,10]. Indeed, IORT was successfully used as an option during lumpectomy in early-stage BC with the goal to deliver high doses directly to the tumor bed, which is considered the site at greatest risk of local recurrence [12]. The direct visualization and positioning of the applicator on the tumor bed led to better local control (LC) and disease-free survival (DFS) after surgical resection, allowing a better intraoperative protection of adjacent healthy structures [13,14]. Beyond the clinical and prognostic advantages, IORT is also beneficial for early-stage BC patients who cannot undergo long and intensive courses of adjuvant external beam (EB)RT due to organizational and/or personal problems, for patients who live at long distances from hospital and for patients with comorbidities not suitable for EBRT [15,16]. In the last two decades, the introduction of dedicated mobile accelerators increased the popularity of intraoperative electron RT (IOERT), and the criteria for patient selection were reviewed in 2020 by the European Society for Radiotherapy and Oncology (ESTRO) [17]. Moreover, the possibility of combination of different IORT treatment modalities as the use of ultrahigh dose rates in a dedicated in-room imaging system can direct to a better optimization of the treatment and consequently to achieve better clinical outcomes [18]. More recently, full-dose IOERT was shown to have non-inferiority compared with whole-breast (WB)RT in terms of cosmesis [19], and excellent LC was reported in an Italian multicenter analysis for anticipated IOERT boost for early-stage BC [20].

Therefore, the aim of this retrospective study was to evaluate clinical response and outcome after full-dose IOERT in patients with early-stage BC treated with conservative surgery in our Radiation Oncology Center at the University Hospital of Ferrara, Italy.

## 2. Materials and Methods

### 2.1. Study Design

This is a retrospective observational study. The study was approved by the Ethics Committee of the University Hospital of Ferrara (IRMA TRIAL 4). All patients gave their written consent.

### 2.2. End Points

The primary objective of the study was to define the clinical response rate after lumpectomy and IORT. The secondary objective of the study was the overall survival (OS) rate and the cosmetic outcome assessment.

### 2.3. Eligibility

Patients with early BC treated with conservative surgery and IOERT from January 2014 to December 2019 were included in the study. Inclusion criteria were as follows: age >60 years, clinical tumor size ≤2 cm (stage ACCJ 7th edition: cT1), luminal A carcinoma (positive hormonal receptors, negative HER2, MIB-1 value <20%), pathological negative lymph nodes (pN0). The exclusion criterion was a lobular carcinoma.

### 2.4. Surgery

All patients were treated with conservative surgery (lumpectomy). The sentinel lymph node was evaluated preoperatively.

### 2.5. Intraoperative Radiotherapy

Full-dose IOERT was delivered using a dedicated mobile accelerator (LIAC 12 MeV model, SIT Sordina IORT Technologies S.p.A., Vicenza, Italy). The main dosimetric characteristics of the electron beams generated by the linear accelerator were recently reported in [21].

A shielding disc was placed under the lumpectomy site to protect adjacent organs at risk (muscles, ribs, lung, also heart and coronary arteries for left breast). Dedicated tube-shaped polymethylmethacrylate (PMMA) applicators with different sizes were used to collimate the electron beams. The applicators were manually attached to the accelerator with a hard docking system. Both flat (0°) and bevelled (15°) applicators were used. For reference flat applicators, the lateral penumbra of the beams ranged from 6 mm (6 MeV) to 10 mm (10 MeV). A planned dose of 21 Gy at depth of the 90% isodose was administered in about 2–3 min. For flat applicators, the depth of 90% of the maximum dose ranged from 16 mm (6 MeV) to 25 mm (10 MeV). After radiation treatment, the lumpectomy cavity was irrigated and the wound sutured.

**Figure 1:**
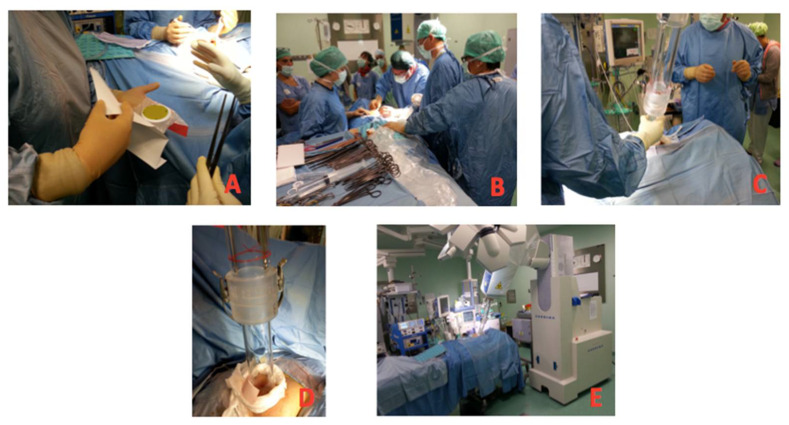
(**A**): preparation of the chest protection disk with dosimetric film; (**B**): measurement of the depth of the breast parenchyma; (**C**): docking maneuver; (**D**): docking done; (**E**): ready for dose delivery.

The surgical time was increased by a mean of 45 min (range: 30–60 min) by the entire IOERT procedure. Figure 1 shows the IOERT process.

### 2.6. Adjuvant Therapy

Patients received adjuvant treatment according to the national cancer guidelines of Italian association of medical oncology (AIOM) [22].

### 2.7. Follow-Up

Clinical, cosmetic and/or instrumental follow-up were performed 45 days after the treatment, 6 months after the first medical check, and every 12 months thereafter. The first evaluation was clinical and included history, physical, ECOG, toxicity, and cosmetic assessment. The subsequent evaluations included clinical and cosmetic evaluation, imaging (mammography and/or breast ultrasound depending on the medical judgment), and laboratory tests.

### 2.8. Statistical Analysis

Acute and late toxicity were assessed with CTCAE v. 4.03 and EORTC-RTOG scales [23,24], respectively. Cosmetic outcome was evaluated using the Harvard/NSABO/RTOG Breast Cosmesis Grading Scale [25]. Survival endpoints (OS and DFS) were analysed with Kaplan–Meier method, using SPSS statistics software (version 29.0, IBM Corp., New York, NY, USA).

## 3. Results

### 3.1. Patients and Treatment Characteristics

A total of 162 women were consecutively treated and analyzed in our Radiation Oncology Center (median age: 71 years, range: 52–89; histology: 129 (79.6%) invasive ductal carcinomas (IDC), 22 (13.6%) mixed histologies, 7 (4.3%) mucinous carcinoma, 2 (1.2%) tubular carcinoma, 1 micropapillary carcinoma (0.6%), and 1 (0.6%) no special type (NST); UICC stage T1a 14 patients (8.6%), stage at surgical specimen T1b 62 patients (38.3%), T1c 76 (46.9%), T2 10 patients (6.2%) with a median dimension of 11 mm, range: 2.5–26 mm). No patient (0.0%) underwent neoadjuvant therapies before surgery. The main patient and tumor characteristics are reported in Table 1. Dedicated IORT applicators with a median diameter of 6 cm (range 5–9 cm) were used to deliver the prescribed dose. The main treatment characteristics are summarized in Table 2.

### 3.2. Response

**Figure 2:**
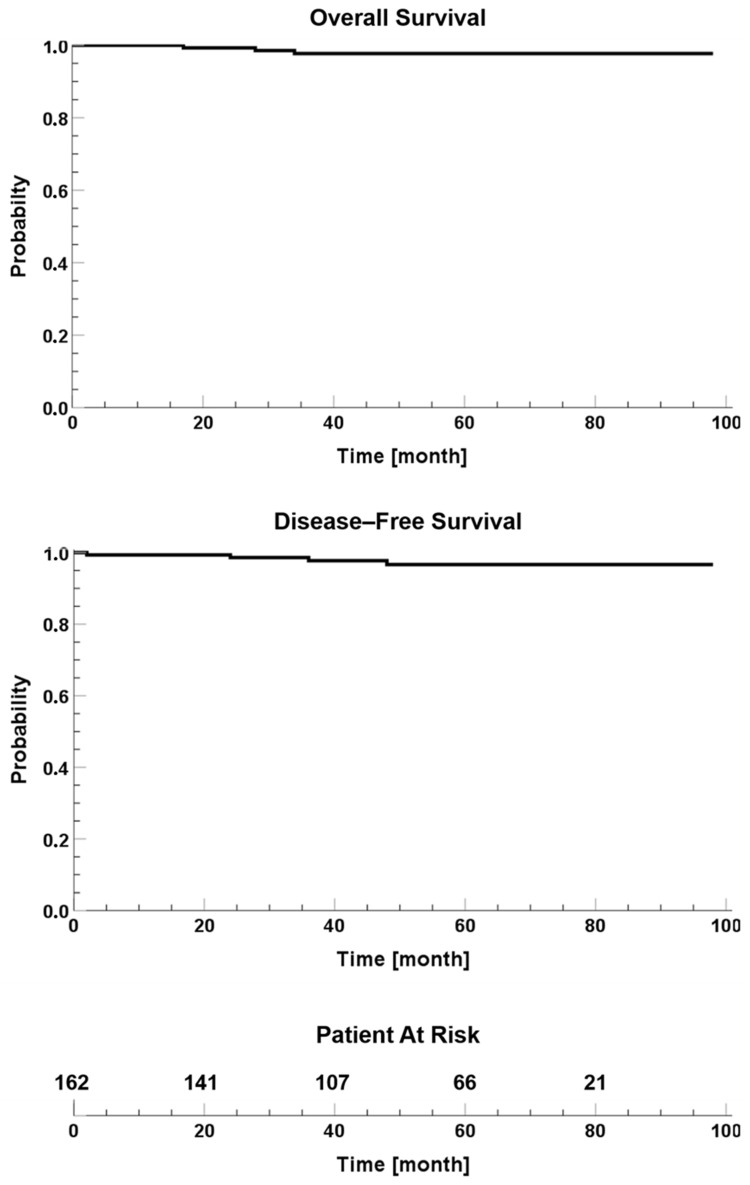
Kaplan–Meier curves of survival endpoints. Top panel: overall survival; central panel: disease-free survival; bottom panel: patient at risk.

A total of 162 consecutive patients were included in this analysis (median follow-up: 54 months, range: 1–98 months). Overall, 114 patients (70.4%) received adjuvant hormone treatment, whereas 48 patients (29.6%) were not eligible for hormone therapy due to very low relapse risk, patient’s drugs intolerance and/or comorbidities, and/or patient’s refusal. In total, 1 patient (0.6%) with adverse histo-pathologic features on final specimen examination (HER2-positive) underwent adjuvant systemic therapy (Taxan and Trastuzumab). Post-IORT clinical and instrumental analyses showed an overall response rate in 97.5% of patients (CI 95%: 93.0–99.0%). Locoregional relapse occurred in 4 patients (2.5%), subsequently treated with mastectomy or re-excision lumpectomy. All of these patients showed ipsilateral breast relapse; only 1 also showed ipsilateral internal mammary lymph node metastasis. Only 1 patient presented disease relapse in the same quadrant (retroareolar region). No patients showed distant metastases. Patients with disease relapse presented a primary tumor histology after lumpectomy of ductal carcinoma (n. 3) and mixed carcinoma (n. 1), primary tumor dimension ranged from 4 to 17 mm (median 10.5 mm), and hormone therapy was recommended to all patients (1 patient refused to take it). The recurrence histologies were: not specific type (NST) infiltrating carcinoma (n. 3) and infiltrating ductal carcinoma (n. 1). The median time to recurrence was 30 months (range: 2–48 months). Five-year OS rates was 98.1%: 3 patients (1.8%) died from comorbidities, none as a consequence of breast cancer. Kaplan–Meier curves for OS and DFS are shown in Figure 2. The number of individuals who were still being followed up at each time point is also reported along the *x*-axis (see bottom panel: “patient at risk”). After treatments, no patients (0%) showed radiation-related acute complications; only a small proportion of patients (17.3%) experienced peri-operative G1 complications such as wound dehiscence, wound infection, and bleeding with hematoma. No patient presented G0-1 late toxicities. Overall, 3.7% of patients showed late moderate/severe tissues fibrosis (5 patients G2 and 1 patient G3 tissues fibrosis). No patient experienced G4 late toxicity. A total of 101 patients (62.3%) showed good cosmetic outcome results, 32 (19.7%) showed fair cosmetic results, and 23 (14.2%) showed excellent cosmetic results. Only 6 patients (3.7%) presented poor cosmetic outcome with marked fibrosis involving more than one-quarter of the breast tissue. Overall, 34 patients (21.0%) were asked for self-evaluation and satisfactory judgment during follow-up, showing a good correlation and agreement between the objective expert panel and subjective patient evaluation. Acute and late toxicity, and cosmetic outcome results are summarized in Table 3.

## 4. Discussion

As the safety and effectiveness of the IOERT-boost was recently confirmed in an Italian multicenter analysis in which our center was included [20], here, we aimed to evaluate the safety and efficacy of full-dose IOERT in our routinely clinical practice for a selected cohort of early BC patients.

In this study, we retrospectively analyzed a series of 162 patients consecutively treated with lumpectomy and IOERT. The treatment was well tolerated with a 5-year overall response rate of 97.5% (CI 95%: 93.0–99.0%) and OS of 98.1%.

The five-year local recurrence and OS rates reported in our study were slightly better in comparison to that showed in the literature as in the phase III ELIOT trial (2.5% versus 4.2%, and 98.1% versus 96.8%, respectively), probably due to a more selective and rigorous patients enrollment excluding those with higher risk characteristics as Grade 3, metastatic axillary lymph nodes, tumor dimension > 2 cm, and triple negative molecular subtype [26,27]. The only risk factor that occurred in our study is the presence at final histopathological specimen of a stage pT2 in 6.2% of patients with a tumor size ranging from 21 to 26 mm Moreover, the randomized phase 3 trial published by Orecchia et al. [26] highlighted ipsilateral breast tumor recurrence outcomes also after 10 and 15 years, showing an increase in the recurrence rate in the IOERT arm (8.1% and 12.6%, respectively). Nonetheless, our data on recurrence rates were slightly unfavorable in comparison to those obtained in adjuvant whole-breast radiotherapy (WBRT) arm in ELIOT trial (5-year rate: 2.5% vs. 0.5%) [26]. However, as our first relapse was observed at only 2 months after surgery plus IOERT suggesting a persistence of the primary disease, for prudential purposes we preferred to consider it as a disease recurrence. On the contrary, Bartelink and collegues showed a 5-year recurrence rate of 7.3% after adjuvant WBRT for stage I-II breast cancer [11]. The TARGIT-A trial showed that low energy X-ray IORT was statistically non-inferior to WBRT with a 5-year local recurrence risk of 2.11% compared to 0.95% postoperative WBRT arm for early BC [28]. Additionally, the study published by Silverstein et al. showed a higher local recurrence rate after kV-IORT over adjuvant WBRT [29]. Despite this, the authors concluded that the higher risk in local relapse must be balanced with patient convenience and compliance, with a minor exposure to medical environments, with a decreased toxicities and complication rates, and at last hospital costs [29]. Additionally, the cosmetic outcome after treatments has to be considered as a factor to take into account in the choice of therapy. In fact, a poor cosmetic outcome may result in a higher prevalence of emotional and psychological disorders in patients [30]. Breast-conserving surgery plus IORT could even reach good or excellent outcomes at 12 months after irradiation in 95% of patients [31]. Moreover, cosmetic results were very good and significantly better after IORT compared with patients treated with IORT + WBRT as in case of adverse histo-pathologic features on the specimen examination (*p* < 0.001) [32]. Our study shows favorable results in the incidence of radiation-induced fibrosis (3.7%) when compared to EBRT with whole-breast irradiation as in the study published by Lyngholm et colleagues (23%) [33]. In the case of cosmetic assessment, the evaluation performed from different points of view is crucial: radiation therapist, radiotherapy nurse and patient. In fact, there is often no complete agreement between the healthcare staff evaluation and that of the patient, despite a similar rating system [34]. In our study, the results on cosmetic outcome were analyzed by a seven-member radiation oncologist panel. Only a small number of patients (21.0%) were asked for a self-evaluation and a subjective satisfactory judgment during the follow-up visit, showing a good correlation and agreement between objective expert panel and subjective patient evaluation.

The present study has some limitations mainly related to its design: the combined treatment (surgery plus radiotherapy) does not allow for a definitive assessment of the impact of IORT alone on acute and late toxicity and on cosmetics, which is hardly attributable to one or the other treatment. However, the irradiation of a small target (surgical bed) compared to what occurs with EBRT, which is delivered to the entire breast tissue, represents an advantage in terms of reduction of radiotherapy-related toxicities. Although all enrolled patients had early BC and were considered to be at low risk of disease recurrence, the administration of hormone therapy also represents an influencing factor for systemic disease control. Furthermore, it would be interesting to evaluate the results in terms of disease-free survival (DFS), OS, toxicity, and cosmetic outcomes even over a longer follow-up time (>5 years). Therefore, our data on long-term treatment tolerance, tumor response, and patient outcomes are only partially reliable. However, all patients were treated homogeneously by the same radiation oncologist and medical physicist team using uniform and standardized patient selection and treatment criteria.

## 5. Conclusions

In conclusion, despite its limitations, our study highlighted the safety and effectiveness of the full-dose IOERT treatment for selected patients. Furthermore, the results presented in this study suggested that the technique and doses used in our experience are able to produce high response rates in disease local control and good cosmetic outcomes. Our data confirmed that careful patient selection is one of the cornerstones in choosing this technique. Therefore, IOERT is currently considered a reasonable option for the treatment of highly selected patients, but further confirmation is needed to definitively consider IOERT as a routinely clinical practice for early BC.

## Figures and Tables

**Table 1:** Main patient and tumor characteristics.

	*n*	%
Patients	162	100
Age		
Median	71	
Range	52–89	
Laterality		
Right	67	41.3
Left	95	58.7
Tumor size [mm]		
Median	11	
Range	2.5–26	
Histology		
IDC	129	79.6
Mixed histologies	22	13.6
Mucinous carcinoma	7	4.3
Tubular carcinoma	2	1.2
Micropapillary carcinoma	1	0.6
NST carcinoma	1	0.6
Tumor stage		
T1a	14	8.6
T1b	62	38.3
T1c	76	46.9
T2	10	6.2
Node stage		
N0	162	100
Grading		
G1	44	27.2
G2	118	72.8
G3	0	0
Luminal A carcinoma	162	100

Abbreviations: IDC = invasive ductal carcinoma; NST = no special type.

**Table 2:** Main treatment characteristics.

	*n*	%
Patients	162	100
Lumpectomy	162	100
IORT	162	100
Applicator [cm]		
5	63	38.9
6	73	45.1
7	22	13.6
8	3	1.8
9	1	0.6
Energy [MeV]		
6	59	36.5
8	68	42.1
10	35	21.4
Prescription dose [Gy]21	162	100
Prescription isodose [%]90	162	100
Adjuvant therapy	115	71.0
Hormone therapy	114	70.4
Chemotherapy	1	0.6

**Table 3:** Acute and late toxicity, and cosmetic outcome results.

Enrolled Patients		No.	%
		162	100
Acute Toxicity	Grade		
-			
Late Toxicity			
Tissues fibrosis	2–3	6	3.7
Cosmetic outcome result			
Excellent		23	14.2
Good		101	62.3
Fair		32	19.7
Poor		6	3.7

## Data Availability

The data presented in this study are available in this article.
